# Effect of Different Cooking Methods on Polyphenols, Carotenoids and Antioxidant Activities of Selected Edible Leaves

**DOI:** 10.3390/antiox7090117

**Published:** 2018-08-30

**Authors:** K. D. Prasanna P. Gunathilake, K. K. D. Somathilaka Ranaweera, H. P. Vasantha Rupasinghe

**Affiliations:** 1 Department of Food Science & Technology, Faculty of Livestock, Fisheries & Nutrition, Wayamba University of Sri Lanka, Makandura, Gonawila, Sri Lanka; 2 Department of Food Science and Technology, Faculty of Applied Sciences, University of Sri Jayewardenepura, Gangodawila, Nugegoda, Sri Lanka; sranaweera@sjp.ac.lk; 3 Department of Plant, Food, and Environmental Sciences, Faculty of Agriculture, Dalhousie University, Truro, NS B2N 5E3, Canada; vrupasinghe@dal.ca

**Keywords:** green leafy vegetables, effect of cooking, polyphenols, antioxidant activity

## Abstract

This study aimed to evaluate the effect of cooking (boiling, steaming, and frying) on polyphenols, flavonoids, carotenoids and antioxidant activity of six edible leaves. The total antioxidant capacity of the fresh and cooked leaves was determined using 2,2-diphenyl-1-picrylhydrazyl (DPPH) radical scavenging and singlet oxygen scavenging assays. The results revealed that frying caused a reduction in major bioactives and antioxidant activities in all leafy vegetables tested. However, steamed and boiled leaves of *C. auriculata* and *C. asiatica* have shown greater levels of polyphenols, flavonoids, and antioxidant capacity compared with fresh leaves. Polyphenol and flavonoid contents of boiled *S. grandiflora* and *G. lactiferum* were higher than that of their fresh form. Boiled and steamed *O. zeylanica* and *S. grandiflora* have shown higher carotenoids. Boiled and steamed leaves of *P. edulis* have shown higher antioxidant activity. The impact of cooking on the changes in bioactive concentrations and antioxidant capacities are dependent on the species and the method of cooking.

## 1. Introduction

The presence and diversity of phytochemicals such as polyphenols, flavonoids, and carotenoids in vegetables are important factors for human health. Many epidemiological studies have shown that the diet rich in antioxidants play an essential role in disease prevention [1] and free radicals are known to be a significant contributor to many degenerative diseases such as cancer, cardiovascular diseases, and diabetes. Dietary antioxidants protect against free radicals such as reactive oxygen species in the human body. Provision of dietary sources of antioxidants that could function to quench or neutralize the spectrum of oxidant sources in the body is important in the prevention of oxidative damage [2]. The green leafy vegetables represent essential nutritional constituents in any balanced diet, and they contain a range of health-related phytochemicals [1]. *Centella asiatica*, *Cassia auriculata*, *Gymnema lactiferum*, *Olax zeylanica*, *Sesbania granadiflora* and *Passiflora edulis* are some of the edible leaves widely consumed as leafy vegetables in Sri Lanka and other tropical countries. These leafy vegetables possess strong antioxidative properties [1,3]. The potential exists for the discovery of synergies between foods such that there would be more than additive effects of consuming the foods in the same meal [2]. Most leafy vegetables can be consumed in their fresh form, and some of them are consumed after being cooked. However, cooking, such as boiling, steaming and frying may cause deterioration of bioactive constituents in kale, cabbage and shallot [4].

Moreover, cooking treatments soften the cell walls and facilitate the extraction of bioactives such as polyphenols [5] and carotenoids [6]. There are published investigations on the influence of cooking methods on the antioxidant activity of some European vegetables [7], African leafy vegetables [8] and brassica vegetables [9], and the different effects reported are dependent on the vegetable types. However, there are limited studies on the effect of domestic cooking on many leafy vegetables available in South Asian origin. Thus, it is important to understand the impact of domestic cooking on antioxidant activity or free radical capacity of leafy vegetables. In the present study, the effect of boiling, steaming, and frying on phytochemical contents (polyphenols, flavonoids and carotenoids) and antioxidant properties (total antioxidant capacity, 2,2-diphenyl-1-picrylhydrazyl (DPPH) radical scavenging ability and singlet oxygen scavenging ability) of six leafy vegetables (*C. auriculata*, *C. asiatica*, *O. zeylanica*, *S. grandiflora*, *G. lactiferum*, and *P. edulis*), were evaluated.

## 2. Materials and methods

### 2.1. Chemicals

All chemicals and solvents used were of analytical grade. Rutin, gallic acid, Folin–Ciocalteu reagent, methanol, *N*-*N*-dimethyl ρ-nitrosoamine, histidine, naphthylethylenediaminedihydrochloride, sodium nitroprusside, 2 deoxy D-ribose were obtained from Sigma Aldrich, St. Louis, MO, USA through Analytical Instrument Pvt Ltd., Colombo, Sri Lanka.

### 2.2. Leaf Samples

Fresh green leafy vegetable samples, *Cassia auriculata* L. (‘Ranawara’), *Olax Zeylanica* (‘mella’), *Centella asiatica* (‘gotukola’), *Gymnema lactiferum* (‘kuringan’-Ceylon cow tree), *Sesbania grandiflora* (‘kathurumurunga’) and *Passiflora edulis* (‘passion fruit’) were collected locally from the Negombo and Makandura areas of Sri Lanka, and they were cleaned and subjected to cooking treatments. Voucher plant specimens from each leafy vegetable collected were deposited in a herbarium.

### 2.3. Cooking Treatments

Three common cooking methods were assessed, such as boiling, steaming, and frying. The cleaned and washed leaves were cut into small pieces, and the samples (400 g) were divided into four parts (100 g each), keeping one portion as control (uncooked, stored at 4 °C in the refrigerator until use for within 24 h), and the rest was subjected to different cooking treatments, such as boiling, steaming and frying, as shown below.

### 2.4. Boiling

Boiling of leaves was conducted as in Jimenez-Monreal et al. [7] with some modifications. Briefly, leaf samples (100 g) were added to boiling tap water (150 mL) in a covered stainless-steel pot and cooked on a moderate flame for 5 min. The samples were drained off and cooled rapidly on plenty of ice.

### 2.5. Steaming

Steaming of leaves was performed as in Miglio et al. [10], with some modifications. Briefly, leafy vegetable samples were placed on a perforated tray in a stainless steel steamer covered over boiling water for 5 min under atmospheric pressure. The samples rapidly cooled on ice.

### 2.6. Frying

Frying of leaves was done as in Jimenez-Monreal et al. [7], with some modifications. Briefly, leafy vegetable samples were added to 500 mL of white coconut oil (Brand name-“N Joy”) in a stainless steel pan at 170 °C and stirred until the sample became crisp-tender. At the end of each trial, samples were drained off and dabbed with blotting paper to allow for the absorption of exceeding oil. After cooking, the leafy vegetable samples were homogenized and stored at −18 °C.

### 2.7. Dry Matter Determination

All the bioactives and antioxidant activities were calculated according to the dry matter basis. Therefore, moisture contents of the cooked samples were determined according to the method described by Turkmen et al. [4].

### 2.8. Preparation of Methanolic Extracts

Methanolic extracts of leaves were prepared according to the method described in Gunathilake et al. [11]. One gram of cooked leafy vegetable samples were weighed and mixed with 8 mL of 70% methanol and vortexed at high speed for thirty minutes and then centrifuged (Hettich, EBA 20, Tuttlingen, Germany) for 10 min at 792× *g*. The extracts were subsequently filtered through a filter paper (Whatman No. 42; Whatman Paper Ltd., Maidstone, UK). The crude extracts were desolventized in a rotary evaporator (HAHNVAPOR, Model HS-2005 V, HAHNSHIN Scientific, Kyonggi-do, Korea) at 40 °C. The concentrated extracts prepared were oven dried at 40 °C for 12 h and were stored at −18 °C in air-tight screw-capped glass vials until assayed within one week. Extracts were dissolved in methanol to obtain a concentration of 3 mg/mL for each assay.

### 2.9. Total Polyphenol Content

The total polyphenol content of the methanolic extracts was estimated by the Folin–Ciocalteu method described by Singleton et al. [12] and with some modifications as described in Gunathilake and Rupasinghe [13]. The concentration of total phenols was expressed as mmol gallic acid equivalents (GAE) per g dry weight (DW) of cooked leaves.

### 2.10. Determination of Total Flavonoid Content

Total flavonoid content was measured according to a colorimetric assay method described in Zhishen et al. [14]. Total flavonoid content in the extracts of green leafy vegetables was expressed as mmol rutin equivalents (RE) per 1 g dry weight of cooked leaf sample.

### 2.11. Determination of Carotenoid Content

The carotenoid content was analyzed according to Türlerinde et al. [15], and carotenoid contents were reported as mg/g DW of the cooked sample.

### 2.12. Total Antioxidant Capacity Assay

The total antioxidant capacity of leaf extracts was analyzed according to Prieto et al. [16]. The antioxidant capacity was expressed as ascorbic acid equivalents (AAE)/g cooked leaves.

### 2.13. Determination of DPPH Radical Scavenging Assay

The capacity of prepared extracts to scavenge the ‘stable’ free radical DPPH was monitored according to Hatano et al. [17]. The percentage inhibition of the radicals due to the antioxidant activity of leaf extracts was calculated.

### 2.14. Singlet Oxygen Scavenging Assay

Singlet oxygen assay was performed according to the method described in Maldonado [18]. The results were calculated as mmol gallic acid equivalent per 1 g of dry weight cooked leaves.

### 2.15. Statistical Analysis

All samples were analyzed in triplicate, and one-way analysis of variance (ANOVA) was performed using MINITAB 15 software (State College, PA, USA). When there were significant differences (*p* > 0.05), multiple mean comparisons were carried out using the least significant difference (LSD) method.

## 3. Results & Discussion

### 3.1. The Effect of Cooking on Total Polyphenol Content

**Figure 1:**
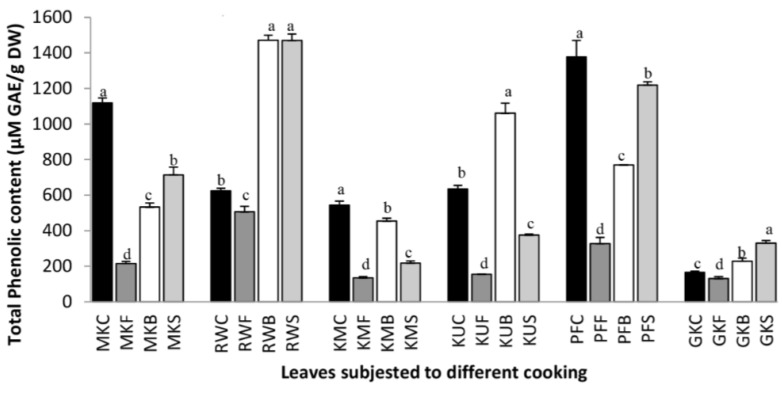
The total phenolic content of raw and cooked extracts of some green leafy vegetables. Values represent the means of triplicate readings. MK, *O. zeylanica*; RW, *C. auriculata*; KM, *S. grandiflora*; KU, *G. lactiferum*; PF, *P. edulis*; GK, *C. asiatica*. C—fresh leaves; F—fried; B—boiled; S—steamed. Data are means ± standard deviations of three replicate determinations. Columns with different letters for each vegetable are significantly different (*p* < 0.05). GAE: gallic acid equivalence; DW: dry weight.

The results of the evaluation of total polyphenols in raw and cooked leafy vegetables are shown in Figure 1. Leaves of *O. zeylanica*, *S. grandiflora*, and *P. edulis* showed the significantly higher (*p* < 0.05) total polyphenol content in their raw samples compared with their cooked samples. This indicates the breaking down of polyphenols in these leafy types during cooking. However, *C. auriculata* leaves showed more than two times higher total polyphenol content in boiled and steamed leaves, compared with its raw form. In previous studies, it was reported that the leaves of *C. auriculata* are rich in polyphenols [1]. *C. asiatica* also showed approximately 200% and 139% higher total polyphenol content in steamed and boiled leaves respectively, compared with its uncooked leaves. *G. lactiferum* showed 167% higher polyphenol content in boiled leaves than in uncooked leaves. This increase in total polyphenols agrees with Turkmen et al. [4], to the extent that the cooking or wet heating could increase the phenol content in some green vegetables such as green beans, broccoli, and pepper. Further, it was reported that the basis of the increase in polyphenol content during cooking could not be categorically stated. However, it could be attributed to the possible breakdown of the complex polyphenolic compounds such as tannins present in the vegetables during heat processing to simple polyphenols [19]. Ferracane and co-authors [5] reported that the increase in total polyphenols during thermal processing might have been due to the liberation of polyphenols from the intracellular protein complexes, changes in plant cell structure, matrix modifications, or the inactivation of the polyphenol oxidases. Moreover, the heat treatments could inactivate polyphenol oxidases, preventing oxidation and polymerization of polyphenols [20]. However, the steamed leaves of *G. lactiferum* showed significantly lower (*p* < 0.05) polyphenol content, only 59% compared with its raw form. Similarly, after the boiling treatment, the total polyphenol content in *P. edulis*, *S. grandiflora*, and *O. zeylanica* have reduced concerning their original content by 55.9%, 83.5%, and 47.6%, respectively. Further polyphenol content in steamed leaves of *P. edulis*, *G. lactiferum*, *S. grandiflora* and *O. zeylanica* also have reduced by 88.5%, 59.1%, 39.9% and 63.8%, respectively with reference to their raw forms. In a previous study, it was reported that blanching of spinach, swamp cabbage, kale, shallots and cabbage for 1 min in boiling water reduced (12–26%) the total polyphenols in these vegetables [4]. However, in a study of Kao et al. [21], a significant increase in total polyphenols were also reported in Thai basil leaf and sweet potato leaf, at the initial stage of boiling for 1 min and 5 min, respectively and subsequently decreased the polyphenol content was also reported as the boiling time increased. By contrast, water cooking has a detrimental effect on polyphenols in vegetables, resulting in a complete loss of polyphenols, likely due to diffusion into the boiling water [10]. In general, frying reduced the polyphenol content in all leafy types studied. Polyphenol content was reduced by 80.7%, 18.9%, 75.2%, 75.8%, 76.3% and 20.6% in *O. zeylanica*, *C. auriculata*, *S. grandiflora*, *G. lactiferum*, *P. edulis* and *C. asiatica*, respectively. In a study of cooked vegetables, it was found that about 60% losses of polyphenols in carrots and broccoli after frying and the losses were higher than during steaming [10]. During deep-fat frying, oils undergo physicochemical changes. Moreover, the food dehydrates, and fat penetrates the food during frying, and therefore food fried in oil may contain higher levels of thermo-oxidized and polymerized products.

### 3.2. The Effect of Cooking on Total Flavonoid Content

**Figure 2:**
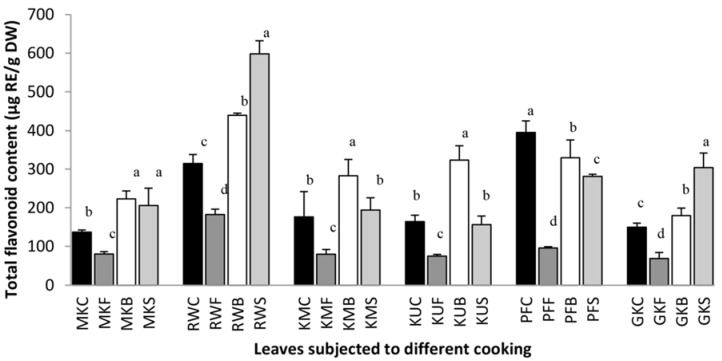
Total flavonoid content of raw and cooked extracts of some green leafy vegetables. Values represent the means of triplicate readings. MK, *O. zeylanica*; RW, *C. auriculata*; KM, *S. grandiflora*; KU, *G. lactiferum*; PF, *P. edulis*; GK, *C. asiatica*. C—fresh leaves; F—fried; B—boiled; S—steamed. Data are means ± standard deviations of three replicate determinations. Columns with different letters for each vegetable are significantly different (*p* < 0.05). RE: rutin equivalance.

Flavonoids, widespread in most common edible fruits, vegetables, and seeds, are heat sensitive polyphenolic compounds. Therefore, the heat exposure during cooking could greatly influence their content in vegetables [22]. Total flavonoid content of raw and cooked leafy vegetables is shown in Figure 2. Total flavonoid content increased significantly (*p* < 0.05) compared with all raw forms after the boiling except *P. edulis*. The percentage increases of flavonoid content in boiled leaves of *O. zeylanica*, *C. auriculata*, *S. grandiflora*, *G. lactiferum* and *C. asiatica* were 163.3%, 140.0%, 160.2%, 197.2% and 120.1% respectively when compared with their fresh leaves. Steamed leaves contain 150.7%, 190.2%, 109.9%, 95.0% and 203.2% flavonoids in *O. zeylanica*, *C. auriculata*, *S. grandiflora*, *G. lactiferum* and *C. asiatica* respectively compared with their raw forms. These increase in flavonoid content after subsequent boiling or steaming may be related to an enhanced availability for extraction, and to a more efficient release of polyphenol or flavonoid compounds from intracellular proteins and altered cell wall structures [9]. Similarly, increased yield of flavonoids in boiled broccoli and spinach was reported previously by Mazzeo et al. [23]. However, in our study, *P edulis* have shown lower flavonoid content after the boiling (83.5%) and steaming (71.2%) with respect to the flavonoid content in its fresh leaves. As in the polyphenols, flavonoid contents in all fried leaves were significantly (*p* < 0.05) lower compared with their fresh contents. However, due to less water solubility of flavonoids in the form of flavonoid glycosides and acylated derivatives, less of these flavonoids are extracted from the plant tissues by the cooking process compared with high soluble glucuronides derivatives [24]. Therefore, most of the flavonoid glycosides and their acylated forms are retained in the tissue during the cooking process [24]. Accordingly, variation in losses and gains of flavonoids due to cooking treatments in the leafy types studied could be due to the types of cooking, the nature of leaves and the forms of the flavonoids present in the plant matrices.

### 3.3. The Effect of Cooking on Total Carotenoid Content

**Figure 3:**
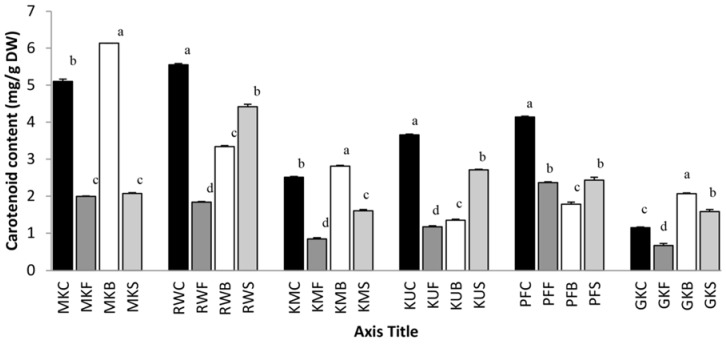
Total carotenoid content of raw and cooked extracts of some green leafy vegetables. Values represent the means of triplicate readings. MK, *O. zeylanica*; RW, *C. auriculata*; KM, *S. grandiflora*; KU, *G. lactiferum*; PF, *P. edulis*; GK, *C. asiatica*. C—fresh leaves; F—fried; B—boiled; S—steamed. Data are means ± standard deviations of three replicate determinations. Columns with different letters for each vegetable are significantly different (*p* < 0.05).

Carotenoid content in raw and cooked leaf samples are shown in Figure 3, and the results showed that the carotenoid contents in all cooked leaves of *C. auriculata*, *G. lactiferum* and *P. edulis* were significantly (*p* < 0.05) lower when compared with their fresh leaves. Steaming of leaves of *O. zeylanica*, *C. auriculata*, *S. grandiflora*, *G. lactiferum* and *P edulis* decreased the carotenoid content by 59.1%, 20.4%, 35.9%, 25.8% and 41.3% respectively in terms of their content in fresh leaves. In frying, carotenoid content of the leaves of *O. zeylanica*, *C. auriculata*, *S. grandiflora*, *G. lactiferum*, *Pedulis* and *C. asiatica* was significantly (*p* < 0.05) reduced by 61.9%, 66.8%, 66.5%, 68.0%, 43.0% and 55.0% respectively, compared to that of their fresh leaves. In some previous studies, a progressive reduction of carotenoids with increasing boiling time in sweet potato was also reported [25]. According to Chen and coauthors [26], the long chains of conjugated carbon-carbon double bonds present in carotenoids are susceptible to light, oxygen, heat and acid degradation. Cooking can also lead to an isomerization from the native all-*trans*-form to its *cis*-isomers [27]. However, from the nutritional standpoint, *cis*-isomers are more bioavailable than all-*trans*-carotenoids in crossing the intestinal wall as they are readily solubilized in micelles [28]. On the other hand, boiling of *O. zeylanica*, *S. grandiflora*, and *C. asiatica* leaves have shown a 19.6%, 11.9% and 79.1% increase in carotenoid content respectively, when compared to their raw contents. Carotenoid content in steamed *C. asiatica* leaves also increased by 37.4%. Some research findings also reported an increase in carotenoids in some green vegetables [29], pumpkin [30] and artichoke [5] after the boiling treatment. According to Khachik and co-authors [31], thermal processing affects the breakdown of the cellulose structure of the plant cells and the denaturation of carotenoid-protein complexes, which allows a more effective and complete extraction of carotenoids. High frying temperatures could, in fact, cause the oil to produce hydroperoxide free radicals and accelerate the degradation of carotenoids [32]. Moreover, frying decreases the initial carotenoid concentration in leafy vegetables, probably because of leaching into oil and to a higher processing temperature. Miglio et al. [10] reported that the carotenoids such as phytoene and phytofluene were completely lost during the frying process. Overall, the results of this study indicating the stability of carotenoids in foods such as leafy vegetables are highly variable.

### 3.4. The Effect of Cooking on Antioxidant Activity

**Figure 4:**
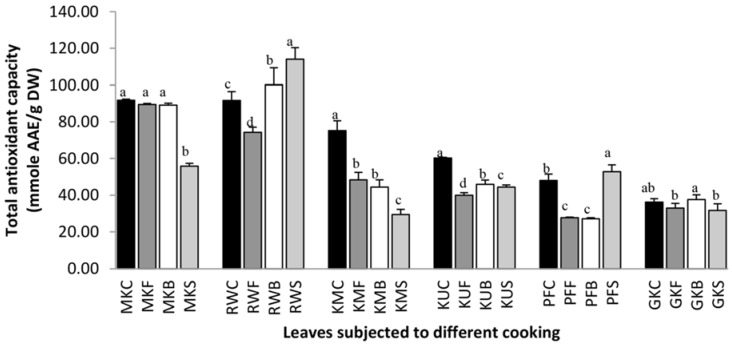
Total antioxidant capacity of raw and cooked extracts of some green leafy vegetables. Values represent the means of triplicate readings. MK, *O. zeylanica*; RW, *C. auriculata*; KM, *S. grandiflora*; KU, *G. lactiferum*; PF, *P. edulis*; GK, *C. asiatica*. C—fresh leaves; F—fried; B—boiled; S—steamed. Data are means ± standard deviations of three replicate determinations. Columns with different letters for each vegetable are significantly different (*p* < 0.05). AAE: ascorbic acid equivalance.

Three different antioxidant activity assays were used to evaluate the effect of cooking on the antioxidant capacity of leafy vegetables. The analytical parameters: linearity, detection limit, precision, and accuracy assay show that the different methods studied are useful for measuring the total antioxidant capacity in foods. This fact is important in order to quantify the changes in the antioxidant capacity of foods during processing/preservation treatment and subsequent storage [33]. Figure 4 displays the total antioxidant capacity of raw and cooked leaf samples. Results clearly show that the total antioxidant capacity of steamed leaves of *O. zeylanica* had significantly (*p* < 0.05) reduced when compared with its raw leaves. However, frying and boiling had not affected the antioxidant capacity of *O. zeylanica* leaves. Although significantly higher (*p* < 0.05) antioxidant capacity was observed in the steamed and boiled leaves of *C. auriculata* compared to that of its fresh leaves, the fried leaves showed lower antioxidant capacity. Significantly lower (*p* < 0.05) antioxidant capacities were observed in all cooked leaves of *S. grandiflora*, and *G. lactiferum* when compared with the antioxidant capacities of fresh leaves. Steamed leaves of *P. edulis* showed 10% higher antioxidant capacity than that of fresh leaves whereas fried and boiled leaves showed nearly 42% lower antioxidant capacities. Boiled *C. asiatica* leaves showed significantly higher (*p* < 0.05) total antioxidant capacity compared to its fresh forms though fried and steamed leaves showed insignificant changes.

**Figure 5:**
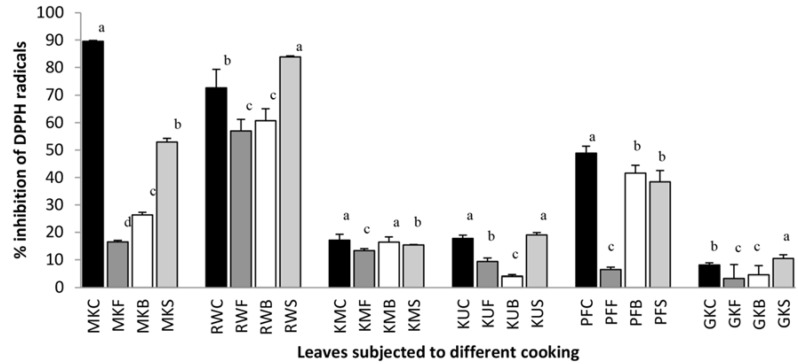
2,2-diphenyl-1-picrylhydrazyl (DPPH) free radical scavenging ability of raw and cooked extracts of some green leafy vegetables. Values represent the means of triplicate readings. MK, *O. zeylanica*; RW, *C. auriculata*; KM, *S. grandiflora*; KU, *G. lactiferum*; PF, *P. edulis*; GK, *C. asiatica*. C—fresh leaves; F—fried; B—boiled; S—steamed. Data are means ± standard deviations of three replicate determinations. Columns with different letters for each vegetable are significantly different (*p* < 0.05).

The radical scavenging activity of leafy vegetables subjected to different cooking treatments was tested using DPPH assay. In this process, polyphenols have the ability to quench DPPH radicals, by providing hydrogen atoms or by electron donation, and to convert them to a colorless product [1]. Hence, the higher the percentage of inhibition of free radical activity, the more potent the antioxidant activity of the extract in terms of hydrogen atom-donating capacity [1]. DPPH radical scavenging ability of raw and cooked leaf samples are shown in Figure 5. In comparison to the scavenging ability of the raw form of studied leafy types, steamed leaves of *C. auriculata*, *G. lactiferum* and *C. asiatica* have shown 15.6%, 1.7% and 29.7% respectively higher scavenging ability, whereas the steamed leaves of *O. zeylanica*, *S. grandiflora*, and *P. edulis* showed 36.7%, 1.7% and 10.5% lower scavenging activity respectively. However, fried and boiled leaves of all leafy types studied showed significantly (*p* < 0.05) lower free radicals scavenging ability when compared with their raw forms. Frying reduced the scavenging ability of *O. zeylanica*, *C. auriculata*, *S. grandiflora*, *G. lactiferum*, *P edulis* and *C. asiatica* by 73.1%, 15.5%, 3.8%, 8.4% 42.5% and 4.8% while boiling of leaves have reduced the free radical scavenging ability by 70.6%, 16.3%, 4.2%, 77.3%, 21.5% and 22.5% respectively. According to our previous findings [1], it was found that the DPPH assay (*r*^2^ = 0.903) and total antioxidant capacity assay (*r*^2^ = 0.890) are correlated with the total phenolic content of leafy vegetable extracts.

**Figure 6:**
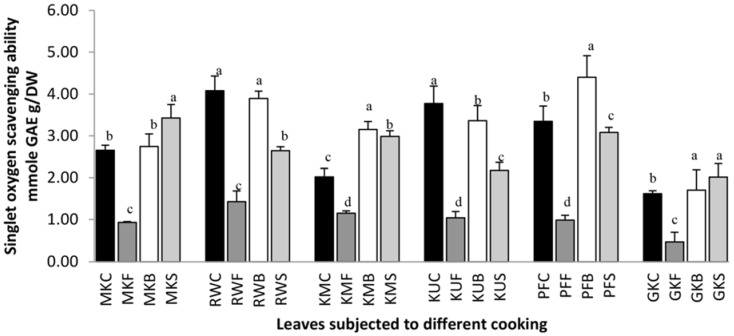
Singlet oxygen scavenging ability of raw and cooked extracts of some green leafy vegetables. Values represent the means of triplicate readings. MK, *O. zeylanica*; RW, *C. auriculata*; KM, *S. grandiflora*; KU, *G. lactiferum*; PF, *P. edulis*; GK, *C. asiatica*. C—fresh leaves; F—fried; B—boiled; S—steamed. Data are means ± standard deviations of three replicate determinations. Columns with different letters for each vegetable are significantly different (*p* < 0.05).

Singlet oxygen is a highly energetic reactive oxygen species which induces a unique oxidation process by directly reacting with electron-rich double bonds without forming free radical intermediates in foods and biological systems [3]. Figure 6 shows the singlet oxygen scavenging ability of raw and cooked leafy types. Results showed that singlet oxygen scavenging properties had reduced during frying in all leaves compared with their fresh leaves and the percentage reduction of *O. zeylanica*, *C. auriculata*, *S. grandiflora*, *G. lactiferum*, *P. edulis* and *C. asiatica* leaves during frying was 64.9%, 65.0%, 43.0%, 72.3%, 70.4% and 71.1% respectively. Boiling of leaves increased the singlet oxygen scavenging ability by 56.0%, 31.4% and 5.4% in *S. grandiflora*, *P. edulis*, and *C. asiatica* respectively. However, boiling has reduced the scavenging activity in *C. auriculata* and *G. lactiferum* by 4.6% and 10.9% respectively and has no significant (*p* > 0.05) effect on *O. zeylanica*. Steaming of leaves has increased the singlet oxygen scavenging activity of *O. zeylanica*, *S. Grandiflora* and *C. asiatica* by 29.1%, 47.9%, and 24.7%, while it has reduced the scavenging ability of *C. auriculata*, *G. lactiferum* and *P. edulis* by 35.1%, 42.3%, and 7.9% respectively, compared with that of their fresh leaves. The decreasing singlet oxygen scavenging activity of leafy vegetables may be due to a reduction in carotenoid content in leaves as carotenoids are effective single scavenging molecules [1]. According to Gunathilake et al. [34], singlet oxygen scavenging ability of leaf extracts correlates with its carotenoid content.

Results showed that some leaves had increased antioxidant activity due to cooking and some leafy types reduced their antioxidant activity after subsequent cooking treatment. Previous studies have shown that the thermal processing of celery [7] increased their antioxidant capacity, after the cooking treatments. It was found that boiling of several vegetables was attributed to the suppression of oxidation by antioxidants due to thermal inactivation of oxidative enzymes [20]. Furthermore, it has been suggested that processing can promote the oxidation of polyphenols to an intermediate oxidation state, which can exhibit a higher radical scavenging efficiency than the non-oxidized polyphenols [35]. Moreover, the boiling process may destroy the cell wall and subcellular compartments, and thus release potent radical-scavenging antioxidants [30]. On the other hand, some leafy vegetables have shown a reduction in their antioxidant activity after the cooking treatments. Some recent studies have also shown a significant reduction in the antioxidant activities after the thermal processing. In another study to determine the effect of thermal processing on the flavonol, rutin, and quercetin, it was shown that the radical scavenging activity decreases with decreasing amount of flavonol in the system [36]. Similarly, Puupponen-Pimia et al. [37] measured the antioxidant capacity by the DPPH assay in blanched cauliflower and reported a reduction of 23%. Overall, the antioxidant activities in various green leafy vegetables were affected differently by thermal processing. The reason could be the differing leafy type’s composition of constituents determining the antioxidant capacity. While some general trends can be observed with previous findings, the effects of processing differ not only according to treatment intensity but also according to the food matrix, suggesting that each matrix should be studied separately [38]. However, it is evident that “antioxidant activity” involves complex interactions amongst intrinsic and extrinsic factors related to the food matrix and organism, which cannot be predicted using simple chemical reactions in a test tube alone and need further analysis [39].

## 4. Conclusions

The findings from the present study indicate that the total polyphenols, carotenoids and antioxidant capacity of selected green leafy vegetables are significantly altered during common cooking practices such as boiling, steaming, and frying. Among the cooking methods evaluated, frying reduces the polyphenols, flavonoids, carotenoids, and antioxidant activities in all leafy vegetables studied, whereas boiling and steaming have shown varying effects on polyphenols, carotenoids, and antioxidant properties, depending on the leafy type. The results of the study can be used for making recommendations on the food processing methods to be chosen for preserving the health benefits of green leafy vegetables.
